# Selective Activation of ET_B_ Receptors Within the Paraventricular Nucleus Attenuates Hypertensive Cardiac Remodelling in DOCA-Salt Hypertensive Rats

**DOI:** 10.3390/ijms272110039

**Published:** 2026-11-09

**Authors:** María Florencia Fernández, Fabián Alejandro Innamorato Costas, Clarisa Marotte, Mónica Navarro, Liliana Graciela Bianciotti, Marcelo Sergio Vatta

**Affiliations:** 1 Instituto de Inmunología, Genética y Metabolismo (INIGEM), Universidad de Buenos Aires (UBA)–Consejo Nacional de Investigaciones Científicas y Técnicas (CONICET), Hospital de Clínicas “José de San Martín”, Av. Córdoba 2351, 9th Floor—Room 6, Buenos Aires 1120, Argentina; mffernandez@docente.ffyb.uba.ar (M.F.F.); marotteclarisa@gmail.com (C.M.); lbianc@ffyb.uba.ar (L.G.B.); 2 Cátedra de Fisiología, Facultad de Farmacia y Bioquímica, UBA, Junín 956, 7th Floor, Buenos Aires 1113, Argentina; fabianinnamoratoc@gmail.com (F.A.I.C.); monicna7@gmail.com (M.N.); 3 Cátedra de Fisiopatología, Facultad de Farmacia y Bioquímica, UBA, Junín 956, 5th Floor, Buenos Aires 1113, Argentina; 4 Instituto de Química y Metabolismo del Fármaco (IQUIMEFA), UBA-CONICET, Junín 956, 7th Floor, Buenos Aires 1113, Argentina

**Keywords:** endothelin B receptor, paraventricular nucleus, DOCA-salt hypertension, cardiac remodelling, sympathetic remodelling, myocardial fibrosis, cardiac hypertrophy, myocardial inflammation

## Abstract

Hypertensive cardiac remodelling results from the interaction of hemodynamic overload, sympathetic dysregulation, and inflammation. The paraventricular nucleus (PVN) is a critical regulator of sympathetic outflow, but the contribution of PVN endothelin B (ET_B_) receptors to cardiac remodelling remains unknown. We investigated whether selective PVN ET_B_ receptor activation protects against cardiac injury in DOCA-salt hypertension. Male Sprague-Dawley rats underwent DOCA-salt hypertension for five weeks and received chronic bilateral PVN infusion of the selective ET_B_ receptor agonist BQ-3020 (52 ng/h) during the final 7 days. Blood pressure and heart rate were monitored longitudinally. Cardiac remodelling was evaluated by echocardiography, histology, immunofluorescence, Western blotting, and RT-qPCR. DOCA-salt hypertension increased PVN ET_A_ and ET_B_ receptor immunoreactivity and produced sustained hypertension, left ventricular hypertrophy, cardiomyocyte enlargement, myocardial fibrosis, and enhanced cardiac sympathetic remodelling, evidenced by increased TH, pTH/TH, and GAP-43. Myocardial TLR4/NF-κB signalling and TNF-α were increased, whereas IL-10 was reduced. PVN ET_B_ receptor activation attenuated blood pressure elevation and markedly reduced hypertrophic, fibrotic, sympathetic remodelling, and inflammatory alterations. BQ-3020 also reduced heart rate AUC in both normotensive and hypertensive rats, without significant changes in the systolic function indices assessed. Selective activation of central ET_B_ receptors attenuates hypertension and cardiac remodelling in DOCA-salt hypertensive rats. These findings support a role for PVN ET_B_ signalling in the modulation of hypertensive cardiac remodelling, with changes in sympathetic remodelling markers suggesting a possible autonomic contribution.

## 1. Introduction

Hypertension remains one of the leading causes of cardiovascular morbidity and mortality worldwide and is a major risk factor for myocardial infarction, heart failure, stroke, and chronic kidney disease [1]. Despite significant advances in antihypertensive therapy, blood pressure remains inadequately controlled in a large proportion of patients, underscoring the need to better understand the mechanisms driving hypertension and its associated target-organ damage [1]. Current evidence indicates that hypertension is a multifactorial disorder characterized by the interplay of neurohumoral activation, immune dysregulation, and chronic inflammation, all of which contribute to progressive cardiovascular injury [2,3].

Among the central mechanisms involved in blood pressure regulation, sustained activation of the sympathetic nervous system is recognized as a major determinant of both hypertension and cardiovascular remodelling [4]. The hypothalamic paraventricular nucleus (PVN) is a key integrative centre that coordinates autonomic, neuroendocrine, and cardiovascular responses through its projections to sympathetic premotor neurons [5,6]. Enhanced neuronal activity within the PVN has been consistently associated with persistent sympathoexcitation in experimental models of hypertension, particularly salt-sensitive hypertension [5,6,7]. Beyond elevating arterial pressure, chronic sympathetic activation directly promotes cardiomyocyte hypertrophy, extracellular matrix remodelling, and ventricular fibrosis, indicating that central autonomic dysregulation is an important contributor to hypertensive cardiac remodelling [4,8].

The endothelin (ET) system is an important regulator of cardiovascular homeostasis in both peripheral tissues and the central nervous system [9,10]. Endothelin-1 (ET-1), the predominant member of this peptide family, acts through two G protein-coupled receptors, ET_A_ and ET_B_, which are widely expressed in autonomic brain regions, including the PVN [9,10]. Central endothelin signalling modulates sympathetic outflow and cardiovascular function, supporting its involvement in the pathophysiology of neurogenic hypertension [10,11].

Most previous studies have focused on the deleterious cardiovascular actions mediated by ET_A_ receptors, including increased sympathetic activity, elevated arterial pressure, vascular dysfunction, and target-organ damage [9,10,11]. In contrast, the physiological and pathophysiological roles of central ET_B_ receptors remain incompletely understood [9,10]. Available evidence suggests that ET_B_ receptor activation may modulate autonomic and cardiovascular function; however, its role within central cardiovascular regulatory pathways remains poorly defined [9,10]. Whether selective activation of central ET_B_ receptors protects the heart from hypertension-induced remodelling has not been established.

Our laboratory has extensively investigated the role of the central endothelin system in cardiovascular regulation. We previously demonstrated that endothelins modulate central noradrenergic neurotransmission under physiological conditions and that this interaction is altered in DOCA-salt hypertension [12,13,14]. More recently, we showed that chronic intracerebroventricular administration of the selective ET_B_ receptor agonist IRL-1620 significantly reduced arterial pressure in DOCA-salt hypertensive rats without affecting blood pressure in normotensive animals and modulated central catecholaminergic signalling [15]. Together, these findings support a role for the central endothelin system in the neurogenic regulation of blood pressure and raise the possibility that selective activation of central ET_B_ receptors may also protect peripheral target organs during hypertension.

Cardiac remodelling is a hallmark of chronic hypertension and results from the combined effects of increased hemodynamic load and sustained neurohumoral activation [8,16]. In addition to pressure overload, persistent sympathetic stimulation amplifies inflammatory signalling and extracellular matrix remodelling, thereby promoting cardiomyocyte hypertrophy and myocardial fibrosis [8,16]. Activation of inflammatory pathways involving Toll-like receptor 4 (TLR4) and nuclear factor-kappa B (NF-κB) further contributes to pathological cardiac remodelling [17]. Consequently, therapeutic strategies targeting central autonomic pathways may simultaneously modulate neural, structural, and inflammatory components of hypertensive cardiac remodelling. Despite accumulating evidence supporting a protective role of central ET_B_ receptor activation in cardiovascular regulation [15], whether this signalling pathway attenuates hypertension-induced cardiac remodelling remains unknown. Moreover, the mechanisms linking ET_B_ receptor activation within the PVN to myocardial structural and inflammatory remodelling have not been elucidated. Therefore, the present study investigated whether chronic activation of ET_B_ receptors within the PVN protects against hypertensive cardiac remodelling in DOCA-salt rats. We assessed whether this intervention lowered arterial pressure and attenuated cardiac hypertrophy, fibrosis, sympathetic remodelling markers, and inflammatory signalling.

## 2. Results

### 2.1. Endothelin Receptor Immunoreactivity in the PVN of Control and DOCA-Salt Animals

Because selective activation of ET_B_ receptors was used as the experimental intervention, we first determined whether the endothelin system was altered within the PVN in DOCA-salt hypertension. Vasopressin immunoreactivity was used to delineate the anatomical boundaries of the PVN and verify the localization of the analysed sections (Figure 1B). Within this nucleus, DOCA-salt hypertensive rats exhibited significantly greater ET_A_ and ET_B_ receptor immunoreactivity than normotensive controls (Figure 1B). Quantitative analysis confirmed significantly increased ETA and ETB receptor immunoreactivity in hypertensive animals (Figure 1A).

### 2.2. PVN ET_B_ Receptor Activation Attenuates the Hemodynamic Alterations Induced by DOCA-Salt Hypertension

To determine whether selective activation of PVN ET_B_ receptors modifies the hypertensive phenotype, systolic blood pressure (SBP) and heart rate were monitored throughout the experimental protocol (Figure 2). DOCA-salt rats exhibited significantly higher SBP than normotensive controls. Chronic bilateral infusion of the selective ET_B_ receptor agonist BQ-3020 into the PVN significantly reduced SBP in hypertensive rats, whereas no changes were observed in normotensive animals. Analysis of the area under the curve for SBP confirmed a significant reduction in the cumulative blood pressure burden in BQ-3020-treated DOCA-salt rats compared with untreated hypertensive animals (Figure 2A). Analysis of heart rate AUC over the treatment period showed a significant reduction in both Control + BQ-3020 and DOCA-salt + BQ-3020 rats compared with their respective vehicle-treated groups (Figure 2B).

### 2.3. PVN ET_B_ Receptor Activation Attenuates Cardiac Structural Remodelling

To determine whether the reduction in arterial pressure induced by PVN ET_B_ receptor activation was associated with changes in cardiac structure, echocardiographic parameters were evaluated (Table 1 and Figure 3). DOCA-salt hypertensive rats exhibited significant increases in left ventricular internal diameter in systole (LVIDs), left ventricular mass (LVM), and left ventricular mass index (LVMI) compared with normotensive controls. Chronic PVN infusion of BQ-3020 significantly reduced all three parameters in hypertensive rats (Table 1 and Figure 3). No significant differences were observed in LV diastolic diameter, ejection fraction, or fractional shortening among the experimental groups (Table 1 and Figure 3).

### 2.4. PVN ET_B_ Receptor Activation Attenuates Cardiac Hypertrophy

To further evaluate the effects of PVN ET_B_ receptor activation on cardiac hypertrophy, the left ventricular hypertrophy index (LVHI), cardiomyocyte cross-sectional area, and the expression of hypertrophic markers were analysed (Figure 4). DOCA-salt hypertensive rats exhibited significant increases in LVHI, cardiomyocyte cross-sectional area, and the protein expression of atrial natriuretic peptide (ANP), B-type natriuretic peptide (BNP), and β-MHC compared with normotensive controls (Figure 4). Chronic activation of PVN ET_B_ receptors significantly reduced LVHI and cardiomyocyte size and markedly attenuated the expression of all hypertrophic markers in hypertensive animals (Figure 4).

### 2.5. PVN ET_B_ Receptor Activation Attenuates Myocardial Fibrosis

To determine whether PVN ET_B_ receptor activation influences myocardial fibrosis, collagen deposition and the expression of profibrotic markers were evaluated (Figure 5). Picrosirius Red staining revealed a marked increase in myocardial collagen deposition, encompassing both interstitial and perivascular compartments, in DOCA-salt hypertensive rats compared with normotensive controls. Chronic bilateral infusion of the selective ET_B_ receptor agonist BQ-3020 into the PVN significantly reduced myocardial collagen deposition in hypertensive animals, whereas no significant effects were observed in normotensive rats (Figure 5A).

Consistent with the histological findings, DOCA-salt hypertension significantly increased the myocardial protein expression of collagen I and collagen III, and the PRO-COL 1 and 3 mRNAs (Figure 5B,C). Chronic PVN ET_B_ receptor activation significantly attenuated the expression of all profibrotic markers in hypertensive rats (Figure 5B,C).

### 2.6. PVN ET_B_ Receptor Activation Attenuates Cardiac Sympathetic Remodelling

To determine whether PVN ET_B_ receptor activation modulates cardiac sympathetic remodelling, tyrosine hydroxylase (TH) phosphorylation and the myocardial distribution of TH- and GAP-43-positive sympathetic fibers were evaluated (Figure 6). DOCA-salt hypertensive rats exhibited a significant increase in the p-THSer40/t-TH ratio compared with normotensive controls (Figure 6A). Chronic PVN infusion of BQ-3020 markedly reduced the p-THSer40/t-TH ratio in hypertensive rats (Figure 6A).

Immunofluorescence analysis further revealed a significant increase in both TH-positive and GAP-43-positive nerve area fractions in the left ventricular myocardium of DOCA-salt hypertensive rats (Figure 6B,C). These changes were markedly attenuated by chronic PVN infusion of BQ-3020 (Figure 6B,C). The coordinated increase in TH-positive sympathetic innervation and GAP-43, a marker of neural plasticity and axonal remodelling, supports the presence of maladaptive cardiac sympathetic remodelling in DOCA-salt hypertension and its attenuation following selective ET_B_ receptor activation within the PVN.

### 2.7. PVN ET_B_ Receptor Activation Modulates Myocardial Inflammatory Signalling

To determine whether PVN ET_B_ receptor activation modulates myocardial inflammatory signalling, components of the TLR4/NF-κB pathway and inflammatory mediators were evaluated in the left ventricular myocardium (Figure 7). DOCA-salt hypertension significantly increased TLR4 protein and mRNA expression, together with increased expression of the pro-inflammatory mediators TNF-α, IL-1β, IL-6, and iNOS (Figure 7A,B). TNF-α, IL-1β and IL-6 mRNA levels were also increased in hypertensive animals (Figure 7B). Chronic PVN ET_B_ receptor activation with BQ-3020 markedly attenuated these inflammatory alterations (Figure 7A,B).

DOCA-salt hypertension was also associated with increased NF-κB/p65 mRNA expression and enhanced myocardial NF-κB/p65 immunoreactivity and nuclear localization, as assessed by NF-κB/p65-DAPI colocalization (Figure 7E,F). These changes were significantly attenuated by chronic PVN ET_B_ receptor activation (Figure 7E,F). In parallel, IL-10 protein expression was reduced and the TNF-α/IL-10 ratio was increased in DOCA-salt hypertensive rats, whereas BQ-3020 restored IL-10 levels and reduced the TNF-α/IL-10 ratio (Figure 7C,D). Together, these findings indicate that PVN ET_B_ receptor activation attenuates the myocardial pro-inflammatory profile associated with DOCA-salt hypertension.

## 3. Discussion

The present study demonstrates that PVN-targeted BQ-3020 administration lowers systolic arterial pressure and attenuates cardiac hypertrophy, myocardial fibrosis, sympathetic remodelling markers, and inflammatory signalling in DOCA-salt hypertensive rats. These findings support a role for central ET_B_ receptor signalling in the modulation of hypertensive cardiac remodelling. Reduced arterial pressure likely contributed to the structural improvements, while the accompanying changes in cardiac sympathetic remodelling markers suggest a possible autonomic contribution that requires functional investigation [8,16]. Together, these observations support the concept that ET_B_ receptor signalling within the PVN modulates the progression of hypertensive heart disease not only by improving systemic hemodynamic but also by limiting neural and molecular processes that drive hypertensive myocardial remodelling.

The increased ET_A_ and ET_B_ receptor immunoreactivity within the PVN indicates an alteration in the local endothelin receptor profile during DOCA-salt hypertension. Enhanced endothelin signalling within autonomic nuclei has been implicated in neurogenic hypertension [10,11,12]. Previous studies in DOCA-salt hypertensive rats reported increased ET_A_ receptor expression without changes in ET_B_ receptors in the posterior hypothalamus [18], suggesting that alterations in the central endothelin system may differ between hypothalamic regions. Our findings extend these observations by demonstrating increased immunoreactivity for both receptor subtypes within the PVN, a key nucleus involved in cardiovascular and autonomic regulation.

The biological significance and underlying mechanisms of the increased ET_A_ and ET_B_ receptor immunoreactivity within the PVN remain to be established. The increased ET_B_ receptor immunoreactivity is consistent with greater abundance of immunodetectable receptor protein but does not directly establish increased receptor functional activity. The parallel increase could reflect shared upstream regulatory influences or distinct mechanisms affecting each receptor subtype. However, receptor mRNA levels and transcription factor activity were not assessed, and the present findings therefore do not establish a common transcriptional mechanism. Changes in receptor synthesis, trafficking, or degradation are additional possibilities that cannot be distinguished by immunofluorescence alone. Furthermore, local endothelin production and availability were not measured; consequently, a compensatory increase in receptor abundance in response to reduced ligand availability cannot be excluded. Our previous findings showing beneficial effects of central ET_B_ receptor activation [15], together with the present response to BQ-3020, support a protective effect of pharmacological activation of this pathway. However, this pharmacological response establishes neither enhanced endogenous ET_B_ signalling nor an adaptive role for the increased receptor immunoreactivity. The interpretation of this increase as an endogenous adaptive response therefore remains a hypothesis requiring further investigation.

The reduction in systolic blood pressure produced by chronic PVN ET_B_ receptor activation likely contributed substantially to the attenuation of cardiac remodelling observed in the present study. Lower arterial pressure decreases ventricular afterload, thereby reducing the mechanical stimulus that drives cardiomyocyte hypertrophy and extracellular matrix remodelling [16,19].

Because the experimental design did not include an antihypertensive comparator producing a similar reduction in arterial pressure, the relative contributions of reduced hemodynamic load and other potential mechanisms cannot be distinguished. Blood pressure lowering may therefore account for a substantial proportion of the improvements in cardiac hypertrophy, fibrosis, sympathetic remodelling markers, and inflammatory signalling. These findings demonstrate that PVN ET_B_ receptor activation attenuates hypertensive cardiac remodelling, although whether this protection includes blood pressure-independent effects remains to be determined.

Alongside the reduction in arterial pressure, PVN ET_B_ receptor activation attenuated changes in tissue markers associated with cardiac sympathetic remodelling. These observations suggest a possible link between hypothalamic autonomic regulation and myocardial remodelling [8,16]. The following sections discuss these findings in relation to the structural and molecular changes observed in the hypertensive myocardium.

The accompanying changes in cardiac sympathetic remodelling markers are consistent with the established involvement of sympathetic mechanisms in blood pressure regulation and hypertensive cardiac remodelling [8,16]. However, the present study cannot determine whether these changes contributed to the cardiac improvements beyond the effects of blood pressure lowering. Cardiac sympathetic remodelling is increasingly recognized as an active contributor to hypertensive heart disease rather than simply a consequence of elevated arterial pressure [8,16]. Beyond sustained adrenergic stimulation, structural and functional alterations of the cardiac sympathetic network may amplify maladaptive neurohumoral signalling and contribute to cardiomyocyte hypertrophy, fibroblast activation, extracellular matrix remodelling, and electrical instability [8,16,20]. Consequently, alterations in cardiac sympathetic remodelling provide a plausible biological framework linking central autonomic dysregulation to progressive myocardial remodelling.

Consistent with this framework, DOCA-salt hypertension markedly increased the myocardial p-THSer40/t-TH ratio together with TH- and GAP-43-positive nerve area fractions. TH is the rate-limiting enzyme in catecholamine biosynthesis, and phosphorylation at Ser40 is a major regulatory mechanism that increases TH enzymatic activity [21]. In parallel, GAP-43 is associated with axonal growth and neural plasticity and has been used to assess myocardial nerve sprouting [22,23]. The coordinated changes in these markers are consistent with alterations in the regulation of catecholamine synthesis and structural remodelling of the cardiac sympathetic network in DOCA-salt hypertension. Their attenuation following PVN infusion of BQ-3020 supports an effect of ET_B_ receptor activation on tissue markers associated with cardiac sympathetic remodelling.

However, these measurements do not directly assess sympathetic nerve activity or outflow, and GAP-43 is not specific to sympathetic nerves. Therefore, the observed changes should not be interpreted as direct evidence of reduced cardiac sympathetic nerve activity. Furthermore, parasympathetic activity and cholinergic innervation were not assessed, and a contribution from parasympathetic modulation cannot be excluded. The reduction in heart rate AUC in both normotensive and hypertensive animals provides additional evidence that BQ-3020 affects cardiovascular regulation. However, this response does not distinguish reduced sympathetic influence from increased parasympathetic influence or other chronotropic mechanisms. Accordingly, the respective autonomic contributions to the effects of PVN ET_B_ receptor activation remain to be determined.

Although the present study does not establish a direct causal relationship between reduced sympathetic remodelling and myocardial protection, the coordinated attenuation of TH phosphorylation and sympathetic neural plasticity strongly supports the concept that ET_B_ receptor signalling within the PVN modulates the cardiac sympathetic network during hypertension [16,21,22,23]. Taken together, these findings identify maladaptive cardiac sympathetic remodelling as a plausible mechanistic link between hypothalamic autonomic regulation and hypertensive myocardial remodelling [8,16].

The attenuation of cardiac sympathetic remodelling was accompanied by a marked improvement in both anatomical and molecular features of cardiac hypertrophy. Chronic PVN ET_B_ receptor activation reduced left ventricular mass and cardiomyocyte cross-sectional area, together with decreased myocardial expression of ANP, BNP, and β-myosin heavy chain (β-MHC). The attenuation of these fetal hypertrophic markers indicates that PVN ET_B_ receptor activation limits not only the structural manifestations of hypertensive hypertrophy but also the associated molecular reprogramming of the myocardium [24,25]. Reduced ventricular afterload and the accompanying changes in cardiac sympathetic remodelling markers provide a physiological context for these structural and molecular findings [8,20,26].

The beneficial effects of PVN ETB receptor activation extended to myocardial fibrosis, as evidenced by reduced myocardial collagen deposition and decreased expression of collagen I and III. Persistent sympathetic activation can promote cardiac fibroblast activation and extracellular matrix remodelling through adrenergic and neurohumoral mechanisms [8,20]. Thus, attenuation of maladaptive cardiac sympathetic remodelling may have contributed to the antifibrotic effects observed in BQ-3020-treated hypertensive rats. Nevertheless, the reduction in arterial pressure also played an important role by decreasing the mechanical and neurohumoral stimuli that promote fibroblast activation and extracellular matrix accumulation [19,20].

The attenuation of cardiac hypertrophy and fibrosis was not accompanied by significant changes in ejection fraction or fractional shortening. Thus, the structural improvements observed in this study were not associated with a detectable improvement in the systolic indices assessed. These findings support attenuation of pathological cardiac remodelling but do not establish improved ventricular performance. Whether the structural changes translate into functional benefits over a longer period remains to be determined. Furthermore, diastolic function was not assessed, precluding conclusions regarding ventricular relaxation, filling, or compliance. The functional significance of the reduced hypertrophy and fibrosis therefore requires further investigation.

Inflammatory activation represents another important component of hypertensive myocardial remodelling and is closely interconnected with neurohumoral and sympathetic mechanisms [2,3,8]. Chronic adrenergic stimulation can promote activation of innate immune and inflammatory pathways within the myocardium, whereas inflammatory mediators may, in turn, enhance sympathetic activity, establishing a reciprocal neuroimmune interaction that contributes to cardiovascular disease progression [8,27]. Consistent with this framework, DOCA-salt hypertension was associated with increased myocardial NF-κB/p65 mRNA expression, enhanced NF-κB/p65 immunoreactivity, and increased nuclear localization, supporting activation of this inflammatory signalling pathway in the hypertensive myocardium. Importantly, chronic PVN ET_B_ receptor activation attenuated these changes while also reducing TLR4 and pro-inflammatory mediator expression and restoring IL-10 levels, resulting in a more balanced myocardial inflammatory profile. The concomitant reduction in TLR4 expression and NF-κB/p65 nuclear localisation is consistent with attenuation of TLR4/NF-κB-associated inflammatory signalling [17,28]. However, these measurements do not establish that this pathway mediates the effects of PVN ET_B_ receptor activation on cardiac remodelling. The inflammatory changes may reflect reduced hemodynamic load or other responses to the intervention. Therefore, the involvement of TLR4/NF-κB signalling should be regarded as an associated molecular finding rather than a demonstrated causal mechanism.

Taken together, the present findings show that PVN-targeted BQ-3020 administration attenuates multiple structural and molecular features of hypertensive cardiac remodelling. Lower arterial pressure and changes in cardiac sympathetic remodelling markers provide a basis for considering hemodynamic and autonomic contributions, but their relative roles and causal relationships remain unresolved. Figure 8 summarises the measured outcomes and distinguishes them from the proposed mechanistic links.

BQ-3020 administration began after four weeks of DOCA-salt exposure, rather than concurrently with hypertension induction. Previous studies have documented ventricular hypertrophy as early as two weeks after DOCA-salt treatment, together with myocardial collagen accumulation that increases between two and four weeks [29,30]. Established cardiac fibrosis after four weeks of mineralocorticoid-salt exposure has also been demonstrated using a separate pretreatment reference group [31]. These observations support the interpretation that our intervention began at a stage when cardiac remodelling would be expected to be already present, although differences between experimental protocols should be considered. However, cardiac structure was not assessed immediately before BQ-3020 administration, and no separate group was evaluated at the four-week time point. Consequently, the endpoint comparisons cannot distinguish regression of pre-existing remodelling from attenuation of further progression, or a combination of both. We therefore describe the observed effects as attenuation of cardiac remodelling rather than demonstrated reversal.

Small sample sizes for some outcomes may limit precision, and generalisability beyond male rats with DOCA-salt hypertension remains to be established. Future studies combining direct measurements of sympathetic nerve activity, an antihypertensive comparator, and receptor-specific interventions would help clarify the mechanisms underlying the observed cardiac effects.

## 4. Materials and Methods

### 4.1. Animals

Male Sprague-Dawley rats (outbred Crl), weighing 125 ± 5 g at the beginning of the experimental protocol, were obtained from the Animal Facility of the Faculty of Veterinary Sciences, University of Buenos Aires (Buenos Aires, Argentina). Animals were allowed to acclimatize for at least 1 week before the initiation of the experimental procedures. Rats were housed in the Animal Facilities of the Department of Physiology, School of Pharmacy and Biochemistry, University of Buenos Aires, and the Institute of Drug Chemistry and Metabolism (IQUIMEFA, UBA-CONICET), under controlled environmental conditions (22 ± 2 °C, 45–55% relative humidity, and a 12 h light/dark cycle), with free access to standard laboratory chow and tap water. No more than four animals were housed per cage. Animal health and welfare were monitored daily throughout the experimental protocol.

Animals were randomly assigned to experimental groups. Whenever feasible, investigators performing data acquisition and quantitative analyses, including echocardiographic, histological, immunofluorescence, and molecular analyses, were blinded to the experimental groups to minimize experimental bias.

All experimental procedures were conducted in accordance with the recommendations of the Guide for the Care and Use of Laboratory Animals of the National Institutes of Health (NIH Publication No. 85-23, revised 1996). The experimental protocol was approved by the Institutional Animal Care and Use Committee of the School of Pharmacy and Biochemistry, University of Buenos Aires (CICUAL-FFyB-UBA; Approval N°. 13082018-160;approval date August 13, 2018; EXP-FYB N°. 0041888/2018; Resolution N°. 3264). Every effort was made to minimize the number of animals used, reduce animal suffering, and implement the principles of the 3Rs throughout the study.

### 4.2. Experimental Design and DOCA-Salt Hypertension Model

Following acclimatization, salt-sensitive hypertension was induced using the deoxycorticosterone acetate (DOCA)-salt model over a 5-week period [14,15]. DOCA-salt hypertensive rats received subcutaneous injections of DOCA (15 mg/kg body weight; Sigma-Aldrich, St. Louis, MO, USA) twice weekly. DOCA was suspended in sesame oil (1 mL/15 mg) and prepared 1 day before each administration. Control animals received equivalent volumes of vehicle. Throughout the experimental protocol, DOCA-salt rats received 1% NaCl as drinking water, whereas control animals received tap water. Standard laboratory chow was available ad libitum to all animals.

At the beginning of the fifth week, animals were randomly assigned to receive bilateral PVN infusion with or without the selective ET_B_ receptor agonist BQ-3020 during the final 7 days of the protocol, generating four experimental groups: Control, Control + BQ-3020, DOCA-salt, and DOCA-salt + BQ-3020. The stereotaxic procedure and chronic bilateral PVN infusion are described in Section 4.3.

Body weight and water intake were recorded three times per week throughout the experimental protocol. Systolic blood pressure and heart rate were monitored at regular intervals as described in Section 4.4.1. Cardiac structure and function were evaluated by transthoracic echocardiography at the end of the treatment period, prior to tissue collection. At the end of the 5-week protocol, animals were euthanized under deep anaesthesia, and tissues were collected for subsequent histological, immunofluorescence, molecular, and biochemical analyses. A schematic representation of the experimental design and timeline is provided in Figure 9.

### 4.3. Stereotaxic Surgery and Chronic Bilateral PVN Infusion

At the beginning of the fifth week of the experimental protocol, rats were subjected to stereotaxic surgery for implantation of bilateral guide cannulas aimed at the PVN. Animals were anesthetized with ketamine/xylazine (50/10 mg/kg, i.p.) and positioned in a stereotaxic apparatus (David Kopf Instruments, Model 900, Tujunga, CA, USA). Bilateral stainless-steel guide cannulas were positioned according to the following stereotaxic coordinates relative to bregma: anteroposterior (AP), −1.8 mm; mediolateral (ML), ±0.5 mm; and dorsoventral (DV), −7.8 mm, according to the rat brain atlas of Paxinos and Watson [32]. The cannula were positioned immediately dorsal to the PVN to minimise direct mechanical injury to the nucleus. BQ-3020 or vehicle was delivered directly through these cannulas, allowing local diffusion towards the PVN.

BQ-3020 (Alomone Labs, Jerusalem, Israel) was continuously infused bilaterally into the PVN using osmotic minipumps (ALZET Model 1004, DURECT Corporation, Cupertino, CA, USA). BQ-3020 was selected for its established ETB receptor selectivity and agonist activity [33]. Additional functional evidence in CNS cells showed that BQ-3020 increased myelin sheath formation in wild-type oligodendrocytes but not in oligodendrocytes with conditional deletion of Ednrb, supporting ETB-dependent activity in that cellular system [34]. Pumps were filled with BQ-3020 (520 ng/µL in artificial cerebrospinal fluid [aCSF]) to deliver 52 ng/h at an infusion rate of 0.11 µL/h for 7 consecutive days. Osmotic pumps were primed in sterile physiological saline at 37 °C for 48 h before implantation according to the manufacturer’s recommendations. The pumps were implanted subcutaneously in the interscapular region and connected to the bilateral guide cannulas.

To minimize the risk of postoperative infection, amoxicillin (100 mg/100 g body weight) was administered orally 1 h before surgery and repeated 48 h later. Following surgery, animals were housed individually throughout the 7-day infusion period.

At the end of the experimental protocol, cannula placement was verified by examining the tracks and tip locations relative to the PVN, using the rat brain atlas of Paxinos and Watson [32] as an anatomical reference. Only animals with confirmed bilateral placement immediately dorsal to the PVN were included in the final analyses.

### 4.4. Experimental Procedures and Functional Assessments

Following completion of the experimental protocol, animals were subjected to the procedures and analyses described below according to the specific experimental endpoint evaluated.

#### 4.4.1. Systolic Blood Pressure and Heart Rate Measurements

Systolic blood pressure (SBP) and heart rate (HR) were monitored throughout the experimental protocol using a non-invasive tail-cuff plethysmography system (NIBP Controller Model 125, ADInstruments, Sydney, Australia) coupled to a PowerLab 8/30 data acquisition system (Model ML870, ADInstruments) and a rat pulse transducer (Model 5160R, Panlab/Harvard Apparatus, Barcelona, Spain).

Animals were acclimated to the experimental environment and measurement procedure for several days before baseline recordings. SBP and HR were measured three times per week during the induction of hypertension and at 24, 48, 72, 96, 120, and 144 h after osmotic minipump implantation. Measurements were performed in a quiet room under controlled environmental conditions and at approximately the same time each morning to minimize circadian variability. To reduce stress-induced variability, animals were gently covered with a soft cloth rather than restrained during the procedure.

For each measurement session, 3–6 consecutive recordings were obtained after signal stabilization, and the mean value was used for statistical analysis. Data were analyzed using LabChart 6.0 software (ADInstruments, Sydney, Australia). SBP was expressed in mmHg and HR in beats per minute (bpm).

For the longitudinal assessment of the cardiovascular response to PVN treatment, SBP and HR values obtained at each time point were analyzed across the 7-day infusion period. The area under the curve (AUC) was calculated for SBP and HR over the treatment period to provide an integrated measure of the cardiovascular response to chronic PVN ET_B_ receptor activation.

#### 4.4.2. Echocardiographic Assessment

Cardiac structure and function were evaluated by transthoracic M-mode echocardiography in five animals per experimental group after 7 days of PVN treatment. Examinations were performed using an EDAN DUS 60 VET ultrasound system equipped with a 7.5 MHz transducer (EDAN Instruments, Shenzhen, China). Animals were anesthetized with urethane (1 g/kg, i.p.) immediately before image acquisition.

The following parameters were assessed: left ventricular internal diameter in diastole (LVIDd) and systole (LVIDs), interventricular septal thickness in diastole (IVSd) and systole (IVSs), left ventricular posterior wall thickness in diastole (LVPWd) and systole (LVPWs), left ventricular mass (LVM), left ventricular mass index (LVMI), relative wall thickness (RWT), endocardial fractional shortening (FS), midwall fractional shortening (MFS), and ejection fraction (EF). Measurements were performed as previously described [35,36].

All echocardiographic examinations and measurements were performed by the same experienced operator, who was blinded to the experimental groups.

#### 4.4.3. Histological and Immunofluorescence Analyses

Confocal Immunofluorescence Analysis of ET_A_ and ET_B_ Receptor Immunoreactivity in the PVN.

At the end of the experimental protocol, rats were deeply anesthetized with urethane (1 g/kg, i.p.) and transcardially perfused with heparinized phosphate-buffered saline (PBS), followed by 4% paraformaldehyde (PFA) in PBS. Brains were removed, post-fixed in 4% PFA for 3 h at 4 °C, cryoprotected sequentially in 15% and 30% sucrose solutions, and stored at −80 °C until processing.

Coronal brain sections containing the PVN were obtained using a cryostat (Leica CM3050 S, Leica Biosystems, Deer Park, IL, USA). Free-floating sections were blocked with 10% normal horse serum containing 0.3% Triton X-100 and incubated overnight at 4 °C with primary antibodies against ET_A_ or ET_B_ receptors together with arginine vasopressin (AVP) as a marker for anatomical identification of the PVN (Table 2). After washing, sections were incubated with the appropriate fluorophore-conjugated secondary antibodies (Table 2) and mounted using Vectashield antifade mounting medium (Vector Laboratories, Burlingame, CA, USA).

Confocal images were acquired using a Zeiss LSM 510 Upright laser-scanning confocal microscope (Carl Zeiss AG, Oberkochen, Germany) equipped with a Plan-Neofluar 25× objective. Images were acquired using identical acquisition settings for all experimental groups, including laser intensity, detector gain, pinhole aperture, optical zoom, and z-step. Quantitative image analysis was performed using ImageJ 1.54f software (NIH, Bethesda, MD, USA) by an investigator blinded to the experimental groups.

##### Histological Assessment of Myocardial Hypertrophy

Following euthanasia, the LV were excised, fixed in 4% PFA, dehydrated through graded ethanol solutions, embedded in paraffin, and sectioned at 3–5 μm.

Sections were stained with hematoxylin and eosin (H&E) according to standard histological procedures. High-resolution images were acquired using an Olympus BX51 microscope equipped with a DP80 digital camera (Olympus, Tokyo, Japan).

Cardiomyocyte cross-sectional area was quantified using ImageJ 1.54f software in transversely sectioned cardiomyocytes displaying a centrally located nucleus. Measurements were performed in randomly selected microscopic fields by an investigator blinded to the experimental groups. Between 150 and 300 cardiomyocytes from 20–30 microscopic fields were analysed per animal. Results were expressed as mean cardiomyocyte cross-sectional area (μm^2^).

##### Histological Assessment of Myocardial Fibrosis

Adjacent LV sections were stained with Picrosirius Red (Sigma-Aldrich, St. Louis, MO, USA) for visualization of myocardial collagen deposition according to standard histological procedures. Images were acquired using the same microscope and imaging conditions employed for H&E-stained sections.

Myocardial collagen deposition was quantified using ImageJ 1.54f software and expressed as the percentage of collagen-positive area relative to the total myocardial area. Quantification included both interstitial and perivascular collagen deposition, without separate analysis of these compartments. Between 20 and 30 microscopic fields were analysed per animal. Quantitative analyses were performed by an investigator blinded to the experimental groups.

##### Immunofluorescence Analysis of Cardiac Sympathetic Remodelling

Paraffin-embedded LV sections were deparaffinized, rehydrated, and subjected to heat-induced antigen retrieval in sodium citrate buffer (0.1 M, pH 6.0). Sections were subsequently permeabilized and blocked with 5% bovine serum albumin (BSA) before overnight incubation at 4 °C with primary antibodies against tyrosine hydroxylase (TH) and growth-associated protein 43 (GAP-43) (Table 2). Sections were then incubated with the corresponding fluorophore-conjugated secondary antibodies (Table 2). Nuclei were counterstained with DAPI-containing Fluoroshield mounting medium (Sigma-Aldrich, St. Louis, MO, USA).

Images were acquired using an Olympus FV1000 laser-scanning confocal microscope (Olympus, Tokyo, Japan) under identical acquisition parameters for all experimental groups. TH- and GAP-43-positive nerve area fractions were quantified using ImageJ 1.54f software following standardized thresholding procedures and expressed as the percentage of positive area relative to the total myocardial area. Quantitative analyses were performed by an investigator blinded to the experimental groups.

##### Immunofluorescence Analysis of Nuclear NF-κB/p65 Localization

LV sections were processed using the immunofluorescence protocol described above and incubated with a primary antibody against NF-κB/p65 (Table 2), followed by the corresponding fluorophore-conjugated secondary antibody. Nuclei were identified by DAPI staining.

Images were analysed using Fiji/ImageJ version 1.54f. The NF-κB/p65 and DAPI channels were separated and converted to 8-bit grayscale images.

For NF-κB/p65 fluorescence intensity analysis, automatic thresholding was performed using the Default method, with the “Limit to threshold” option enabled. The entire image was selected to measure the mean gray value of pixels within the threshold range. A background region without detectable specific staining was also selected, and its mean gray value was measured using the same threshold settings and measurement option. A background-normalised intensity index was calculated as the mean gray value of threshold-selected pixels in the image divided by that of the background region, multiplied by 100.

NF-κB/p65 nuclear localisation was assessed through its spatial association with DAPI using the Colocalization Colormap plugin, with the Autothreshold option enabled. The correlation index (Icorr) generated by the plugin represents the fraction of pixels exhibiting positive correlation between the fluorescence intensities of the two channels, calculated from the normalised mean deviation product (nMDP).

Between 20 and 30 microscopic fields were analysed per animal. Control sections were processed without antibodies to assess autofluorescence or with secondary antibody alone to assess nonspecific staining. For each animal, intensity indices and Icorr values were averaged separately for immunostained images and corresponding controls. The respective mean control value was subtracted from the mean value of immunostained images. Control-corrected Icorr values were additionally multiplied by 100 and expressed as percentages. Each analysis yielded one final value per animal. Quantification was performed by an investigator blinded to experimental group allocation.

#### 4.4.4. Western Blot Analysis

Left ventricular tissue samples were homogenized, and protein extracts were prepared for Western blot analysis. Protein samples were separated by SDS-PAGE and subsequently transferred onto polyvinylidene difluoride (PVDF) membranes (GE Healthcare, Amersham Biosciences, Little Chalfont, UK). Membranes were blocked with 5% non-fat dry milk and incubated overnight at 4 °C with the corresponding primary antibodies (Table 3). After washing, membranes were incubated with the appropriate horseradish peroxidase (HRP)-conjugated secondary antibodies for 2 h at room temperature (Table 3).

Protein bands were visualized using a chemiluminescent Western blot detection reagent (Kalium Technologies, Buenos Aires, Argentina) and recorded on X-ray film (Carestream Health, Rochester, NY, USA). Densitometric analysis was performed using ImageJ 1.52a software. Target protein signals were normalized to GAPDH as the loading control, except for phosphorylated TH (p-THSer40), which was normalized to total TH. Data were expressed relative to the Control ± SD.

#### 4.4.5. RNA Isolation and Quantitative Reverse Transcription Real-Time PCR (RT-qPCR)

Total RNA was isolated from left ventricular tissue using TRI Reagent^TM^ (Molecular Research Center INC, Cincinnati, OH, USA) according to the manufacturer’s instructions. Genomic DNA contamination was removed by treatment with RQ1 RNase-Free DNase (Promega, Madison, WI, USA) for 40 min at 37 °C. RNA concentration and integrity were assessed by UV spectrophotometry and 1% agarose gel electrophoresis, respectively.

Complementary DNA (cDNA) was synthesized by reverse transcription using MMLV reverse transcriptase (Promega), oligo-dT primers, dNTPs, and RNase inhibitor (Genbiotech, Buenos Aires, Argentina). Successful cDNA synthesis was verified by conventional PCR, and target specificity was confirmed by agarose gel electrophoresis based on the presence of a single amplification product of the expected size.

Quantitative real-time PCR was performed using left ventricular cDNA in reaction mixtures containing GoTaq G2 DNA polymerase and the corresponding reaction buffer (Promega), dNTPs, MgCl_2_, and EvaGreen fluorescent dye (Biotium, San Francisco, CA, USA). Primer sequences and thermocycling conditions are provided in Table 4. Gene expression levels were normalized to the housekeeping gene GAPDH, and relative expression was calculated using the 2^−ΔΔCT^ method [37].

### 4.5. Statistical Analysis

Statistical analyses were performed using GraphPad Prism 8.0 (GraphPad Software, San Diego, CA, USA). Data are presented as mean ± standard deviation (SD). Data distribution was assessed using the Shapiro–Wilk test.

For comparisons between two experimental groups, an unpaired Student’s *t*-test was used, with Welch’s correction applied when variances were unequal. For comparisons among more than two experimental groups, one-way analysis of variance (ANOVA) followed by Tukey’s multiple-comparisons test was used.

Longitudinal changes in systolic blood pressure (SBP) and heart rate (HR) during the 7-day PVN treatment period were analysed using two-way repeated-measures ANOVA, with experimental group as the between-subject factor and time as the within-subject factor, followed by Tukey’s multiple-comparisons test. The integrated cardiovascular response over the treatment period was additionally assessed by calculating the area under the curve (AUC) for SBP and HR using the trapezoidal method. A *p* value < 0.05 was considered statistically significant.

## 5. Conclusions

PVN-targeted BQ-3020 administration lowers arterial pressure and attenuates cardiac hypertrophy, fibrosis, sympathetic remodelling markers, and myocardial inflammation in DOCA-salt hypertensive rats. These findings support a role for central ET_B_ receptor signalling in hypertensive cardiac remodelling. The relative contributions of blood pressure lowering and other potential mechanisms remain to be established, and the changes in sympathetic remodelling markers do not directly demonstrate reduced sympathetic nerve activity. Direct bilateral infusion targeting the PVN remains an experimental approach, and the present findings do not establish the clinical feasibility of targeting this pathway.

## Figures and Tables

**Figure 1:**
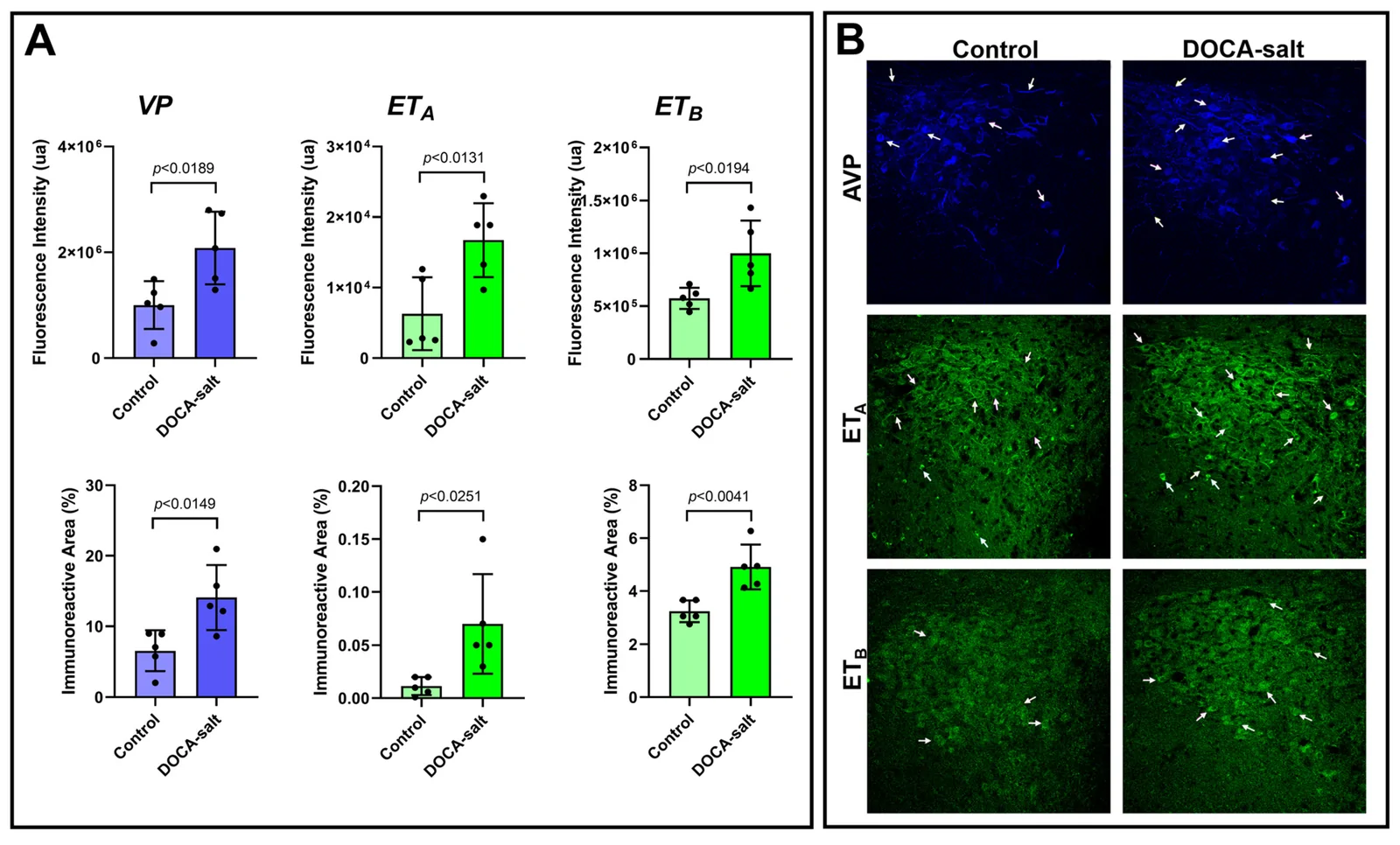
Increased vasopressin, ET_A_, and ET_B_ receptor immunoreactivity in the paraventricular nucleus of DOCA-salt rats. (**A**) Quantitative analysis of vasopressin (AVP), ET_A_, and ET_B_ immunoreactivity in the paraventricular nucleus (PVN) of control and DOCA-salt rats. Fluorescence intensity and immunoreactive area were quantified for each marker. (**B**) Representative immunofluorescence images showing AVP, ET_A_, and ET_B_ immunoreactivity in the PVN of control and DOCA-salt rats. AVP immunoreactivity was used to delineate the anatomical boundaries of the PVN and confirm the localization of the analysed sections. Arrows indicate immunoreactive structures. Data are presented as mean ± SD. Number of animals per experimental group: 5.

**Figure 2:**
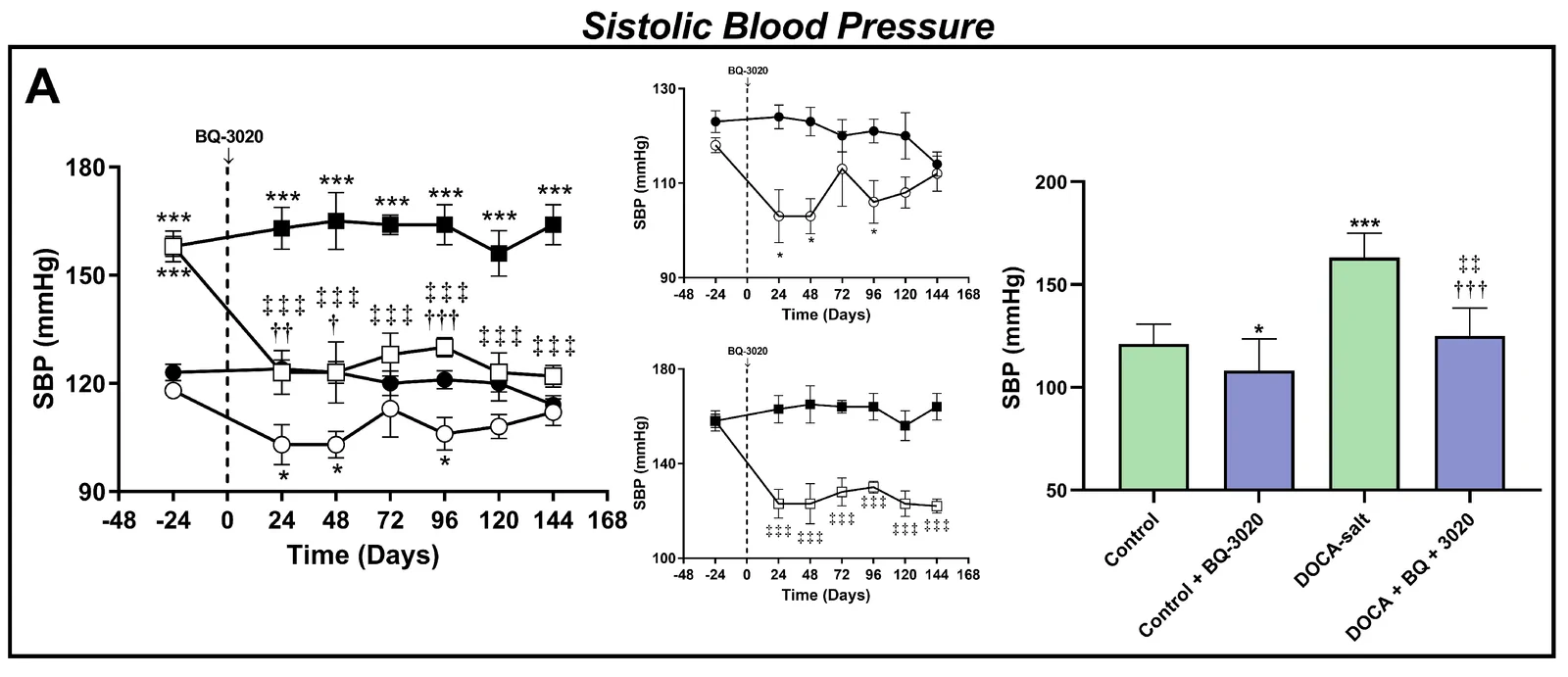

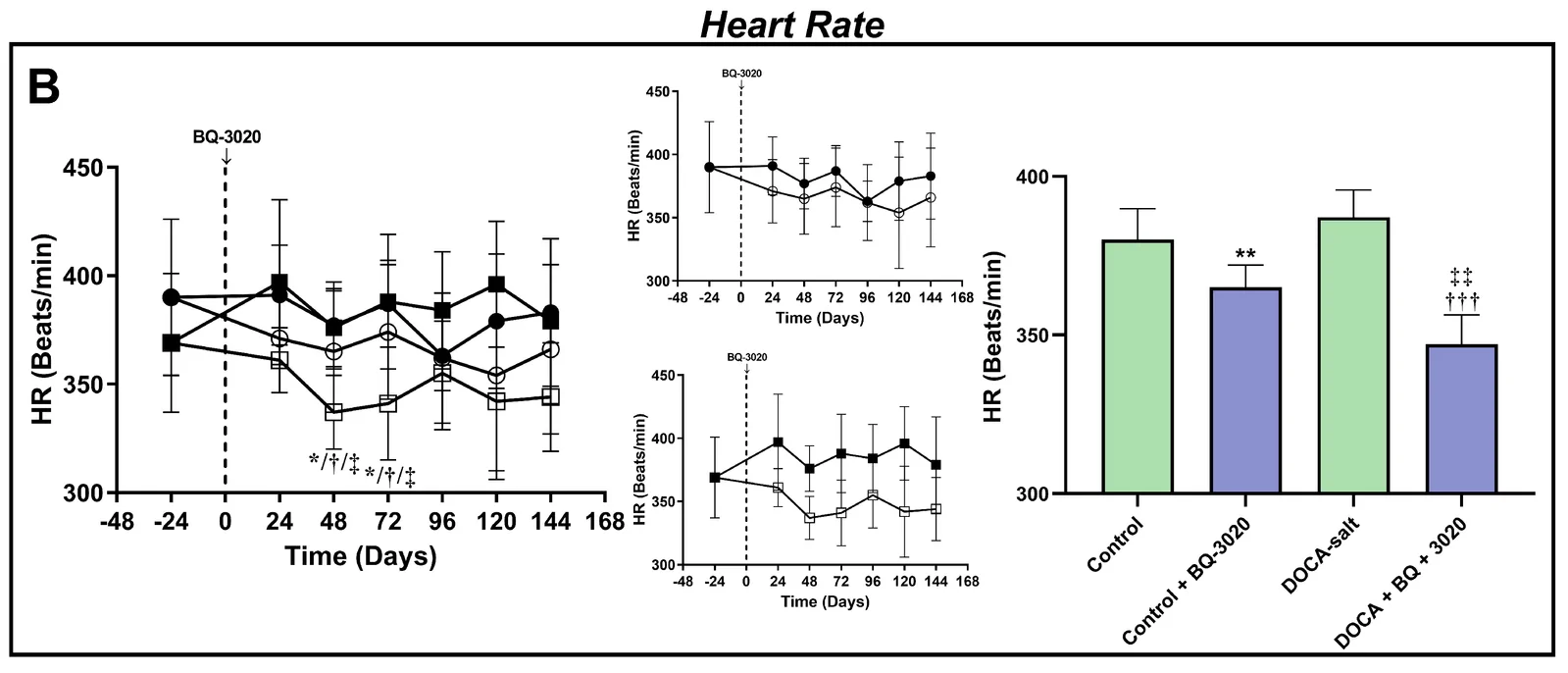
Modulation of systolic blood pressure and heart rate during chronic ET_B_ receptor activation. (**A**) Time course of systolic blood pressure (SBP, mmHg) during chronic infusion of the ET_B_ receptor agonist BQ-3020 from 24 to 144 h. Values recorded during the 24 h preceding treatment are shown as baseline reference for all experimental groups. The overall SBP response during the treatment period was evaluated by calculating the area under the curve (AUC) between 24 and 144 h. (**B**) Time course of heart rate (HR, beats per minute, bpm) during chronic infusion of BQ-3020 from 24 to 144 h. Values recorded during the 24 h preceding treatment are shown as baseline reference for all experimental groups. The overall HR response during the treatment period was evaluated by calculating the AUC between 24 and 144 h. * *p* < 0.05, ** *p* < 0.01 and *** *p* < 0.001 vs. Control; † *p* < 0.05, †† *p* < 0.01 and ††† *p* < 0.001 vs. DOCA-salt; ‡ *p* < 0.05, ‡‡ *p* < 0.01 and ‡‡‡ *p* < 0.001 vs. Control + BQ-3020. Number of animals per experimental group: 14 (Control), 13 (Control + BQ-3020), 13 (DOCA-salt), and 11 (DOCA-salt + BQ-3020). Data are presented as mean ± SD.

**Figure 3:**
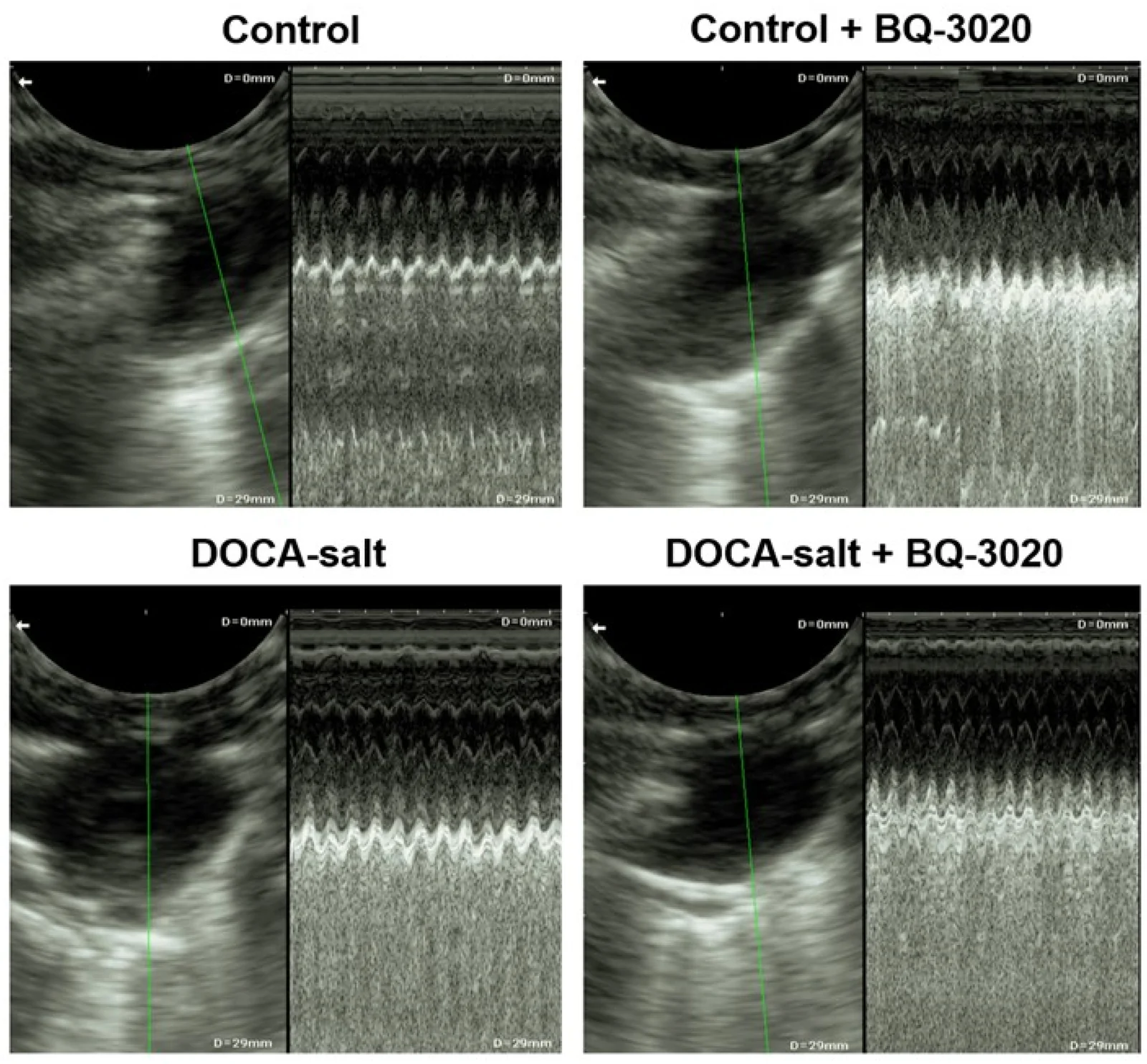
Representative echocardiographic images of the left ventricle in the four experimental groups. Representative M-mode echocardiographic images of the left ventricle. The green line and arrow are guides used to read and measure the echocardiographic images.

**Figure 4:**
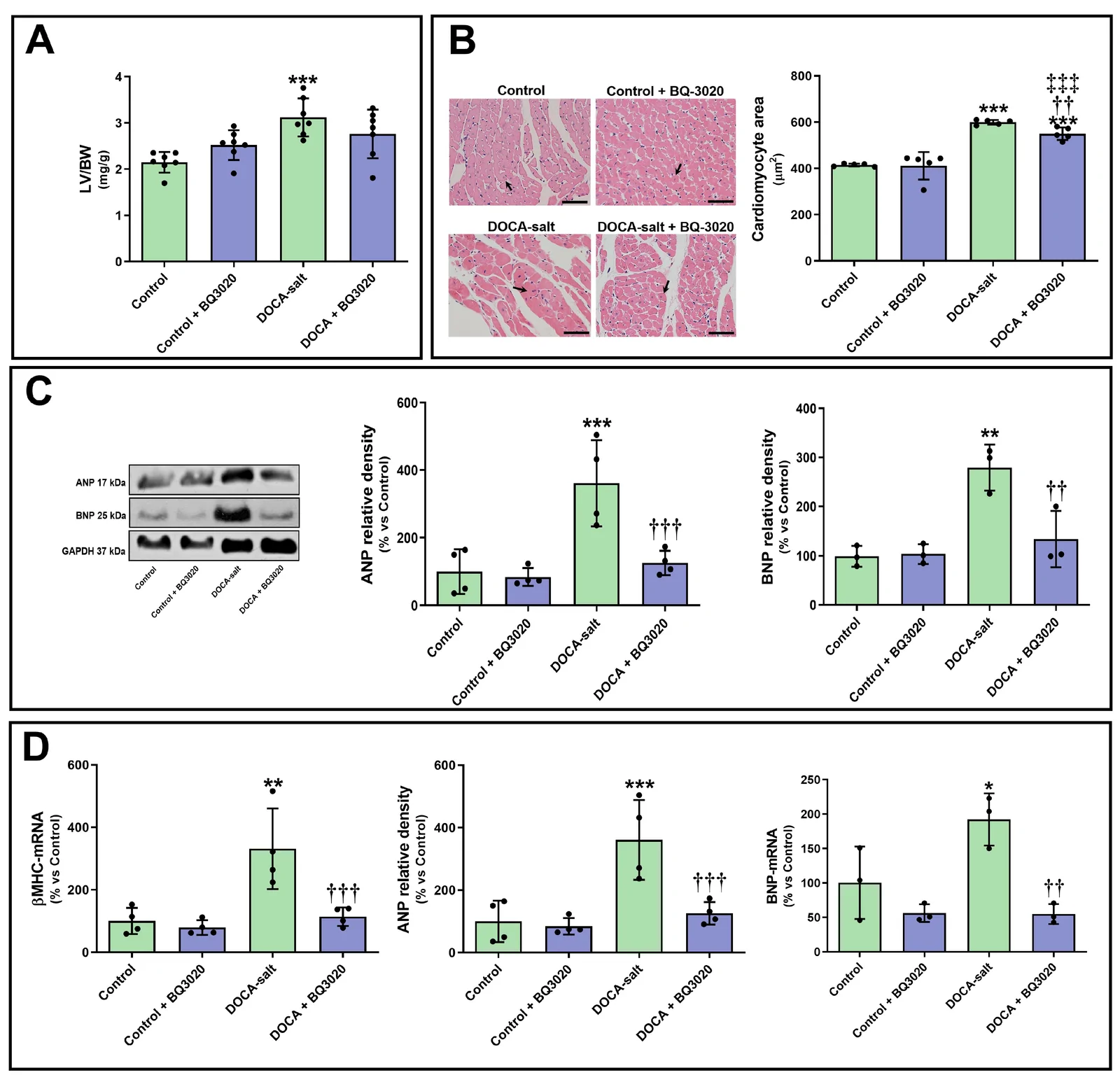
Effects of chronic ET_B_ receptor activation on left ventricular hypertrophy in DOCA-salt rats. (**A**) Left ventricular weight-to-body weight ratio (LV/BW, mg/g) (*N* = 7). (**B**) Representative hematoxylin and eosin (H&E)-stained sections of the left ventricle and quantitative analysis of cardiomyocyte cross-sectional area (*N* = 6). Arrows indicate representative cardiomyocytes used for cross-sectional area assessment. (**C**) Representative Western blots and densitometric analysis of atrial natriuretic peptide (ANP) and B-type natriuretic peptide (BNP) protein expression in the left ventricle (*N* = 4 and 3, respectively). GAPDH was used as a loading control. (**D**) Relative mRNA expression of β-myosin heavy chain (β-MHC), ANP, and BNP in the left ventricle, as determined by RT-qPCR (*N* = 4, 3 and 3, respectively). * *p* < 0.05, ** *p* < 0.01 and *** *p* < 0.001 vs. Control; †† *p* < 0.01 and ††† *p* < 0.001 vs. DOCA-salt; ‡‡‡ *p* < 0.001 vs. Control + BQ-3020. Data are presented as mean ± SD.

**Figure 5:**
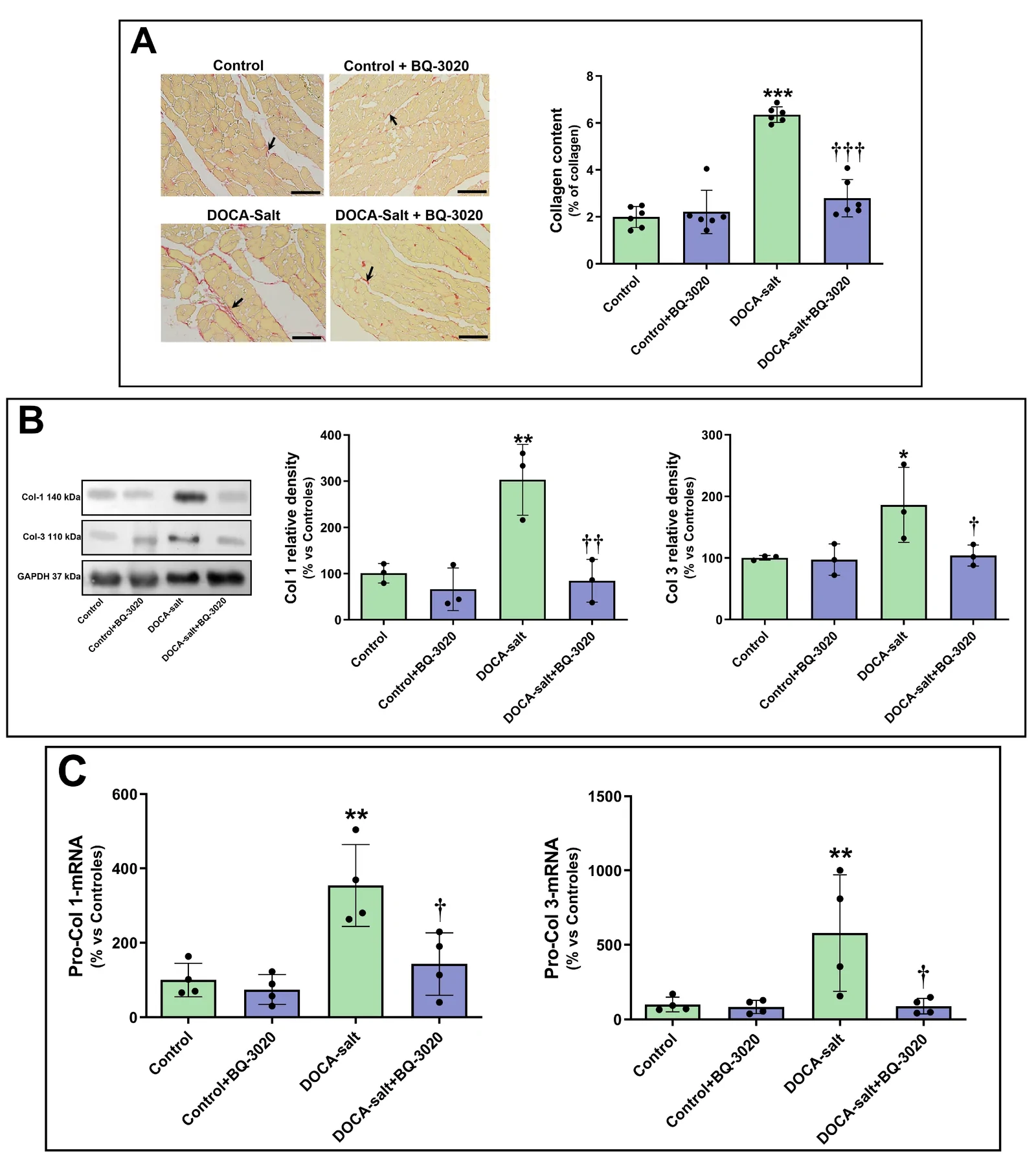
Effects of chronic ET_B_ receptor activation on left ventricular fibrosis in DOCA-salt rats. (**A**) Representative Picrosirius Red-stained sections of the left ventricle and quantification of myocardial collagen area fraction (*N* = 6). Quantification included both interstitial and perivascular collagen deposition, without separate analysis of these compartments. Arrows indicate areas of collagen deposition. (**B**) Representative Western blots and densitometric analysis of collagen type I (COL I) and collagen type III (COL III) protein expression in the left ventricle (*N* = 3). GAPDH was used as a loading control. (**C**) Relative mRNA expression of procollagen type I (Pro-Col I) and procollagen type III (Pro-Col III) in the left ventricle, as determined by RT-qPCR (*N* = 4). * *p* < 0.05, ** *p* < 0.01 and *** *p* < 0.001 vs. Control; † *p* < 0.05, †† *p* < 0.01 and ††† *p* < 0.001 vs. DOCA-salt. Data are presented as mean ± SD.

**Figure 6:**
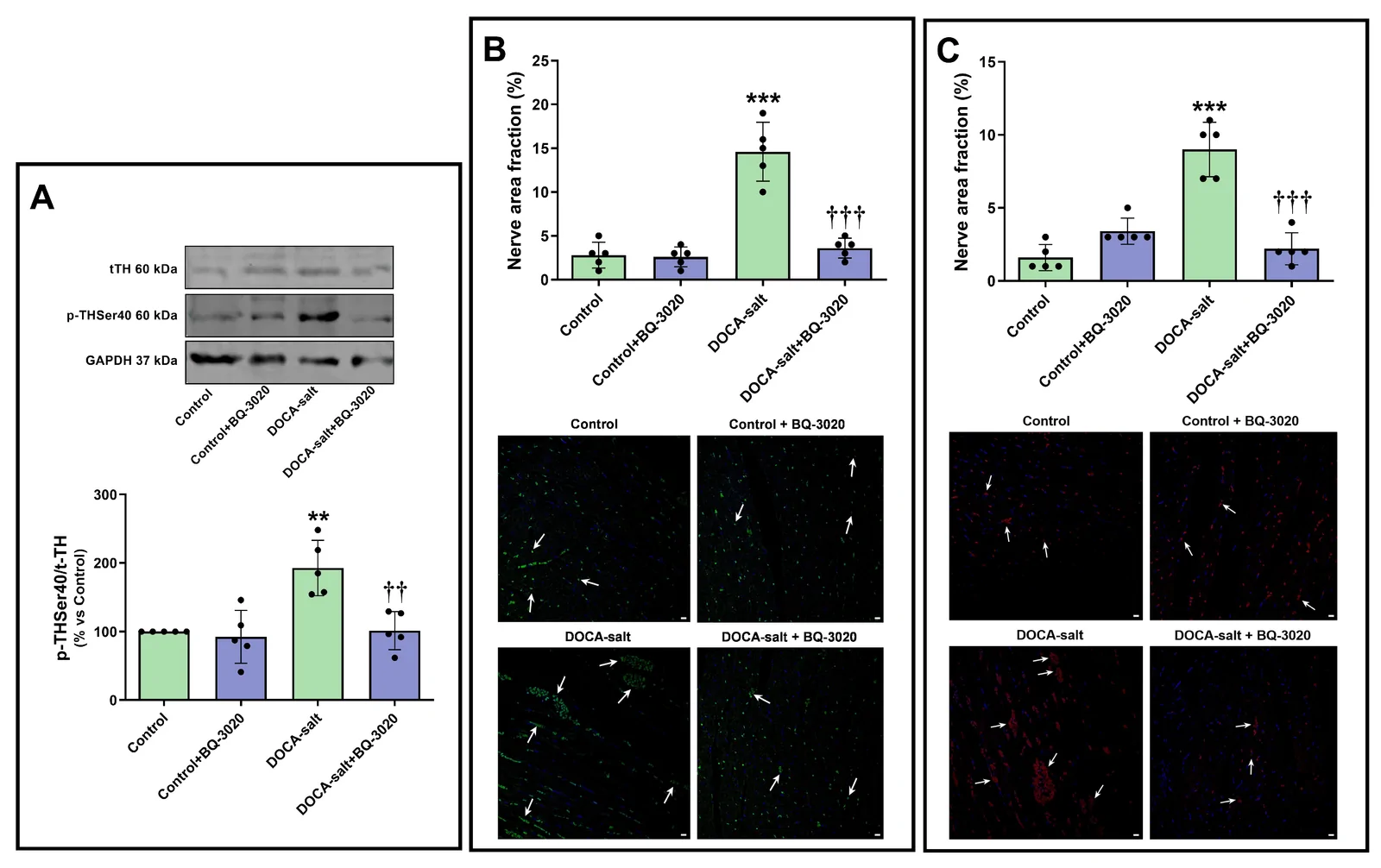
Effects of chronic ET_B_ receptor activation on cardiac sympathetic innervation in DOCA-salt rats. (**A**) Representative Western blots and densitometric analysis of tyrosine hydroxylase (TH) phosphorylation at Ser40, expressed as the p-THSer40/t-TH ratio, in the left ventricle (*N* = 5). (**B**) Representative immunofluorescence images of TH-positive sympathetic nerve fibers in the left ventricle and quantitative analysis of TH-positive nerve area fraction (%) (*N* = 5). Arrows indicate TH-positive nerve fibers. (**C**) Representative immunofluorescence images of growth-associated protein 43 (GAP-43)-positive nerve fibers in the left ventricle and quantitative analysis of GAP-43-positive nerve area fraction (%) (*N* = 5). Arrows indicate GAP-43-positive nerve fibers. ** *p* < 0.01 and *** *p* < 0.001 vs. Control; †† *p* < 0.01 and ††† *p* < 0.001 vs. DOCA-salt. Data are presented as mean ± SD.

**Figure 7:**
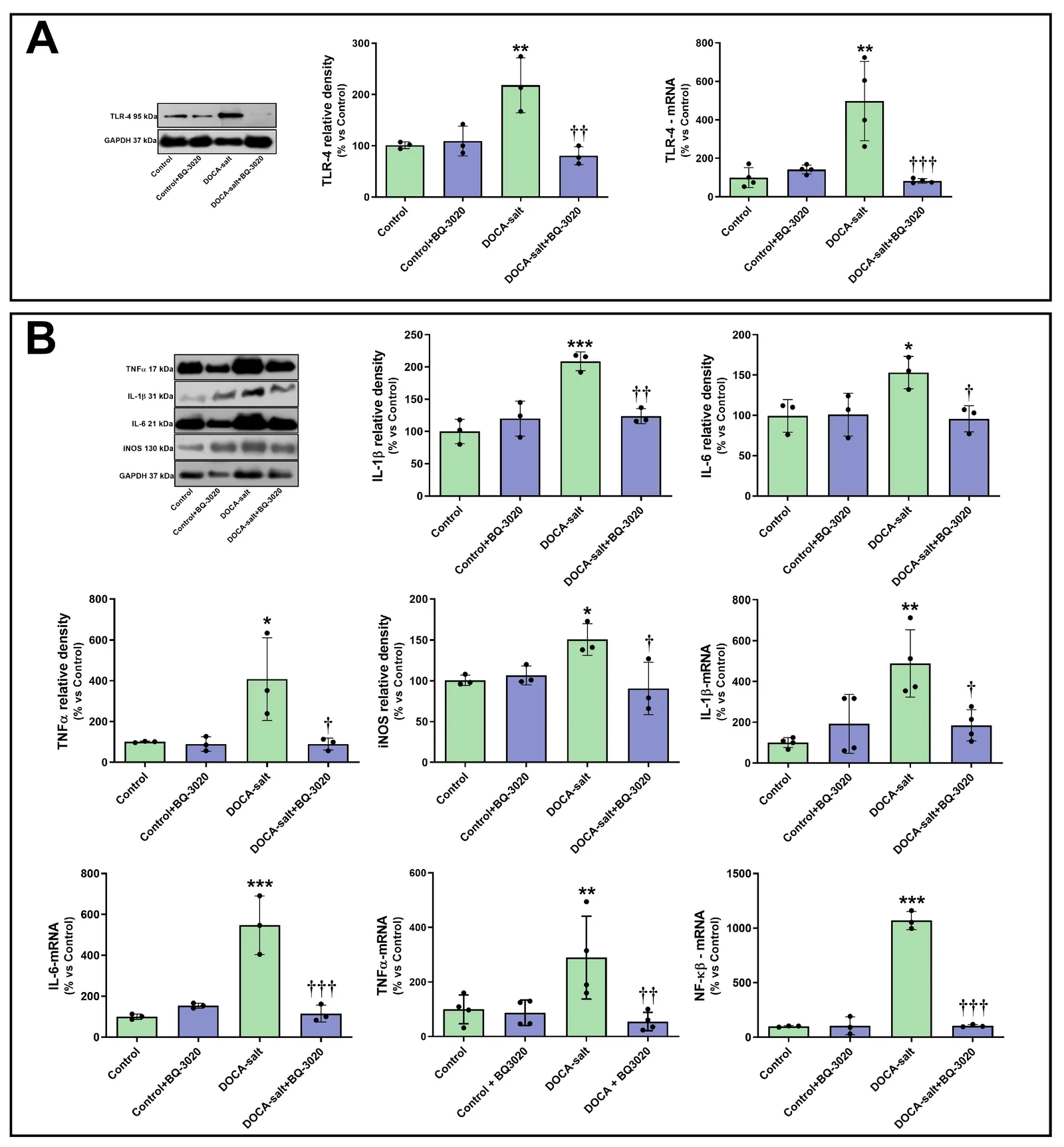

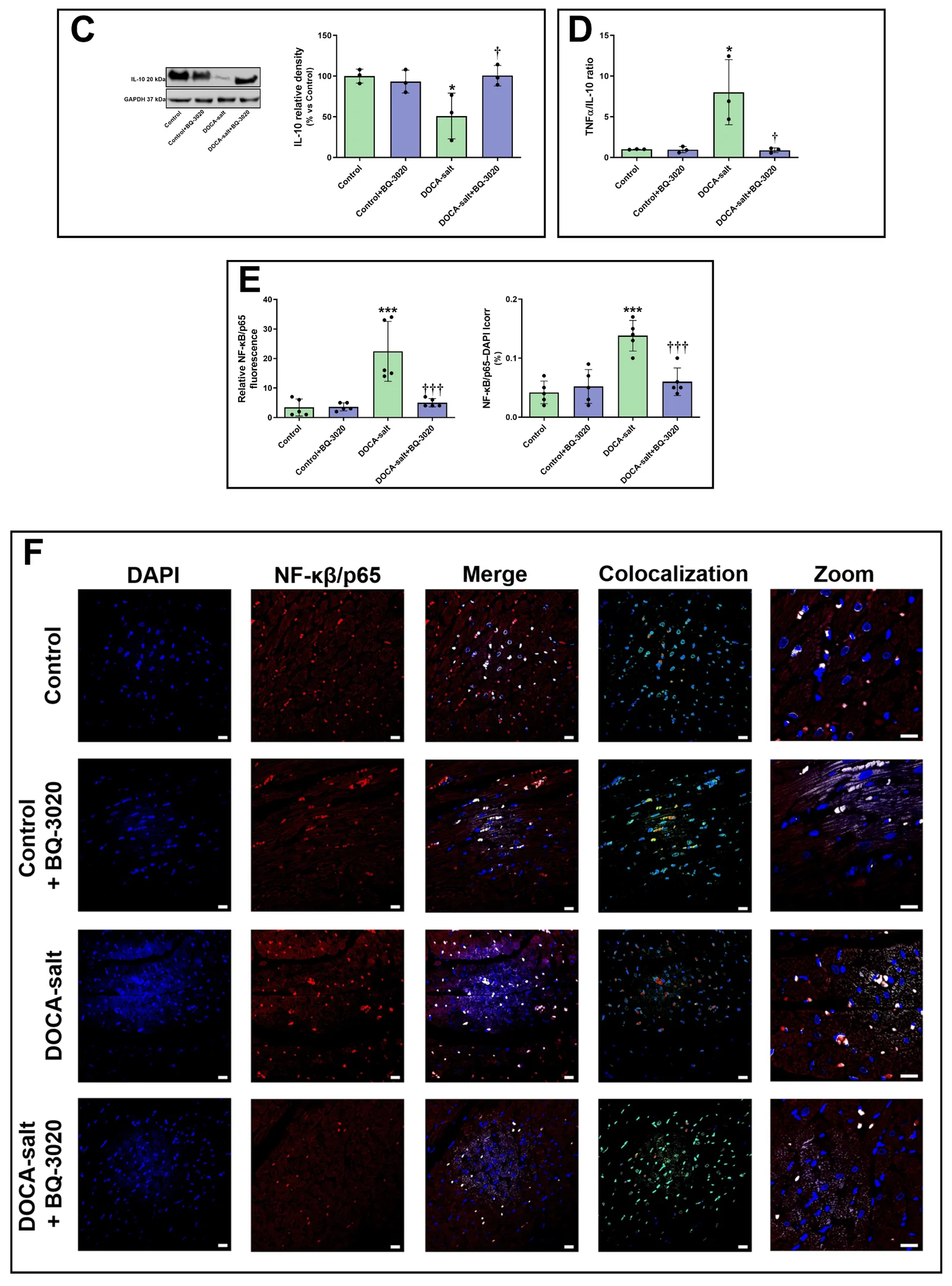
Effects of chronic ET_B_ receptor activation on left ventricular inflammatory signalling in DOCA-salt rats. (**A**) Representative Western blot and densitometric analysis of Toll-like receptor 4 (TLR4) protein expression, together with relative TLR4 mRNA expression in the left ventricle (*N* = 3 and 4, respectively). GAPDH was used as a loading control. (**B**) Representative Western blots and densitometric analysis of tumor necrosis factor-α (TNF-α), interleukin-1β (IL-1β), interleukin-6 (IL-6), and inducible nitric oxide synthase (iNOS) protein expression in the left ventricle (*N* = 3–4). GAPDH was used as a loading control. Relative mRNA expression of IL-1β, IL-6, and nuclear factor kappa B (NF-κB) was determined by RT-qPCR (*N* = 3–4). (**C**) Representative Western blot and densitometric analysis of interleukin-10 (IL-10) protein expression in the left ventricle (*N* = 3). GAPDH was used as a loading control. (**D**) TNF-α/IL-10 protein expression ratio (*N* = 3). (**E**) Quantitative analysis of relative NF-κB/p65 fluorescence intensity and NF-κB/p65–DAPI colocalization in the left ventricle (*N* = 5). Relative fluorescence was calculated as the ratio of mean image to background intensities within the threshold range and corrected by subtracting the corresponding mean staining-control value. Colocalization was expressed as the control-corrected correlation index (Icorr) (%). (**E**) Quantitative analysis of relative NF-κB/p65 fluorescence intensity and NF-κB/p65–DAPI colocalisation in the left ventricle (*N* = 5). Relative fluorescence was calculated as the ratio of mean image to background intensities within the threshold range, multiplied by 100 and corrected by subtracting the corresponding mean staining-control value. Colocalisation was expressed as the control-corrected correlation index (Icorr) multiplied by 100. (**F**) Representative immunofluorescence images of NF-κB and DAPI staining used for quantitative analysis. * *p* < 0.05, ** *p* < 0.01 and *** *p* < 0.001 vs. Control; † *p* < 0.05, †† *p* < 0.01 and ††† *p* < 0.001 vs. DOCA-salt. Data are presented as mean ± SD.

**Figure 8:**
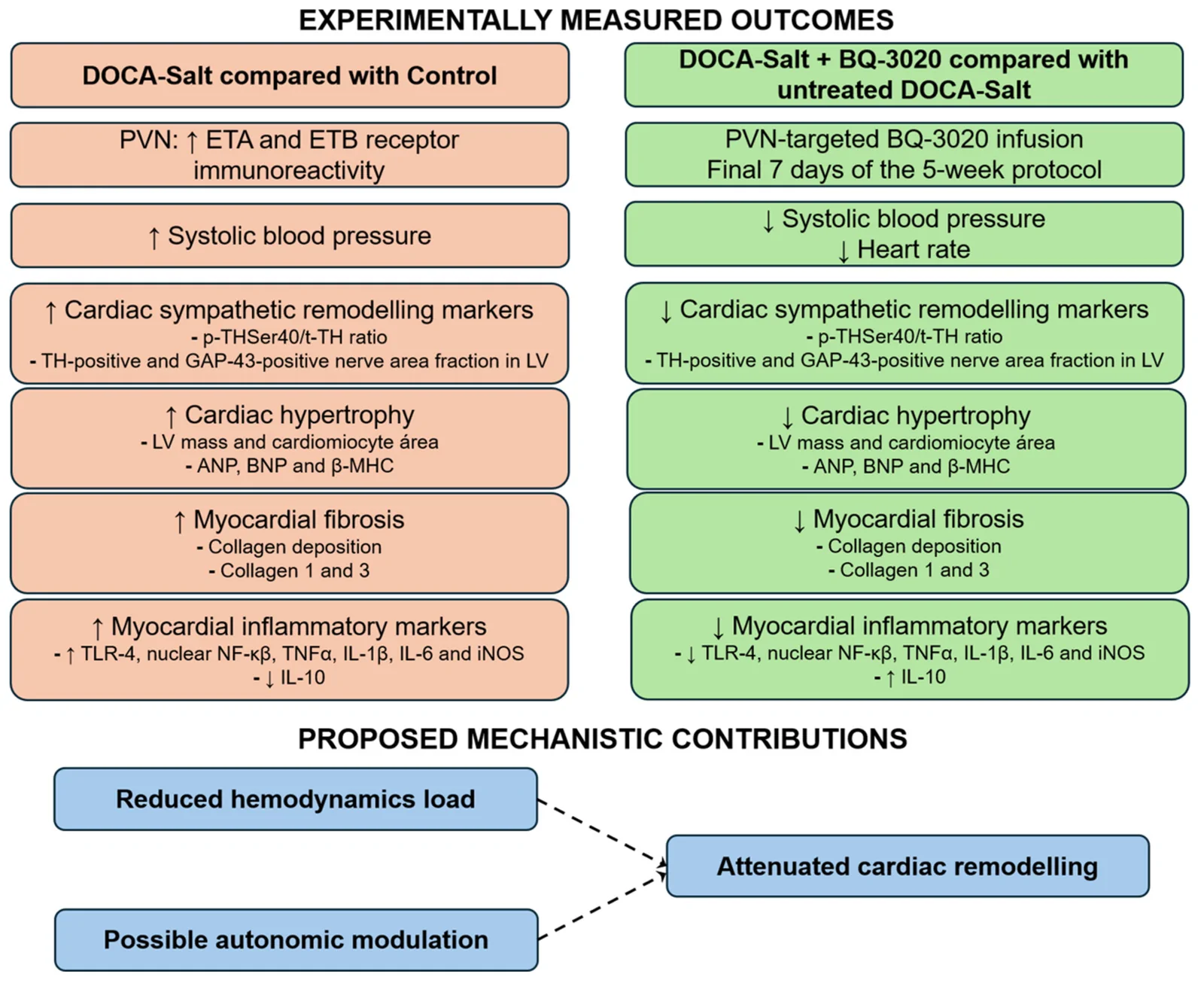
PVN-targeted BQ-3020 administration attenuates hypertensive cardiac remodelling in DOCA-salt rats: summary of experimental findings and proposed mechanistic links. DOCA-salt hypertension increased PVN ET_A_ and ET_B_ receptor immunoreactivity and systolic arterial pressure, accompanied by cardiac hypertrophy, myocardial fibrosis, and alterations in sympathetic remodelling and inflammatory markers. BQ-3020 administration during the final seven days of the five-week protocol lowered systolic arterial pressure, heart rate and attenuated these cardiac alterations, including reductions in the p-THSer40/t-TH ratio, TH-positive and GAP-43-positive fibre area fractions, hypertrophic indices, collagen deposition, and pro-inflammatory markers, together with increased IL-10. The upper panels summarise measured outcomes; upward and downward arrows indicate differences relative to Control animals in the left panel and untreated DOCA-salt animals in the right panel. The lower section depicts reduced hemodynamic load and possible autonomic modulation as proposed contributors to the attenuation of cardiac remodelling; dashed boxes and arrows distinguish these mechanistic hypotheses from the measured outcomes. PVN, paraventricular nucleus; TH, tyrosine hydroxylase; LV, left ventricular.

**Figure 9:**
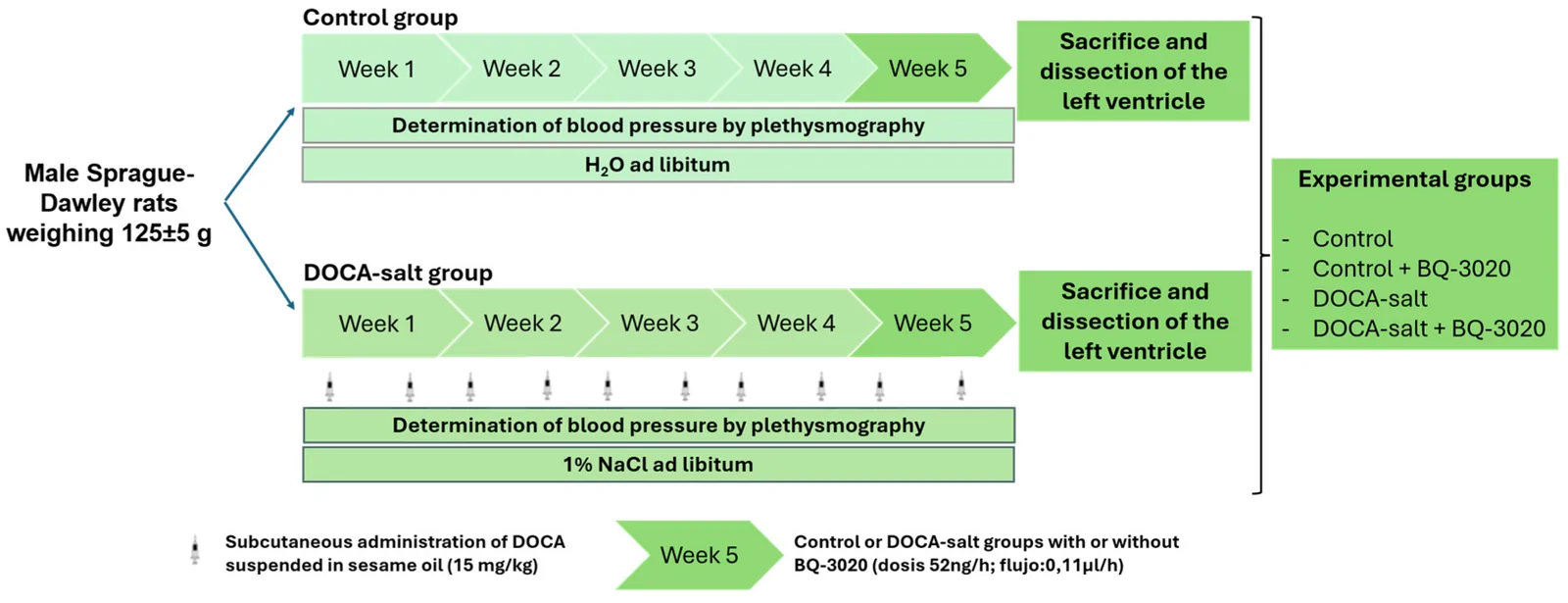
Schematic representation of the experimental protocol. Male Sprague-Dawley rats were subjected to the Control or DOCA-salt protocol for 5 weeks. DOCA-salt hypertension was induced by subcutaneous administration of DOCA acetate (15 mg/kg, twice weekly) together with 1% NaCl solution provided ad libitum throughout the experimental period, whereas Control rats received water ad libitum. Blood pressure was monitored by plethysmography during the protocol. During the fifth week, BQ-3020 was continuously infused bilaterally into the paraventricular nucleus (PVN) for 1 week (52 ng/h; infusion rate, 0.11 μL/h) using osmotic minipumps, resulting in four experimental groups: Control, Control + BQ-3020, DOCA-salt, and DOCA-salt + BQ-3020. At the end of the 5-week experimental period, animals were euthanized and the left ventricle was dissected for subsequent analyses.

**Table 1:** Echocardiographic Parameters. Abbreviations: EF, ejection fraction; FS, endocardial fractional shortening; IVSd, interventricular septal thickness in diastole; IVSs, interventricular septal thickness in systole; LVIDd, left ventricular internal diameter in diastole; LVIDs, left ventricular internal diameter in systole; LVM, left ventricular mass; LVMI, left ventricular mass index; LVPWs, left ventricular posterior wall thickness in systole; MFS, midwall fractional shortening; RWT, relative wall thickness.

Parameter	Control	Control + BQ-3020	DOCA-Salt	DOCA-Salt + BQ-3020
**LVIDd (mm)**	4.55 ± 1.12	4.66 ± 1.24	4.49 ± 1.09	4.26 ± 1.25
**LVIDs (mm)**	2.86 ± 1.18	2.33 ± 1.48	1.90 ± 0.90	1.86 ± 1.23
**IVSd (mm)**	1.14 ± 0.09	1.16 ± 0.26	1.39 ± 0.26	1.21 ± 0.28
**IVSs (mm)**	2.31 ± 0.20	2.32 ± 0.20	2.97 ± 0.21 **	2.44 ± 0.28 **^††^**
**PWd (mm)**	2.39 ± 0.63	2.81 ± 0.73	4.03 ± 0.81 *	3.07 ± 0.75
**PWs (mm)**	3.24 ± 0.44	3.45 ± 0.94	4.52 ± 0.69 *	3.58 ± 0.61
**LVM (mg)**	470.55 ± 220.87	680.44 ± 138.00	1052.67 ± 162.97 ***	579.83 ± 217.26 **^††^**
**LVMI (mg/g)**	1.18 ± 0.60	1.97 ± 0.42	2.68 ± 0.56 **	1.65 ± 0.66 **^†^**
**FS (%)**	53.04 ± 16.31	54.21 ± 17.95	50.61 ± 10.51	59.34 ± 13.24
**MFS (%)**	19.11 ± 3.42	21.30 ± 6.18	22.70 ± 3.11	24.11 ± 2.09
**RWT**	1.04 ± 0.26	1.16 ± 0.61	1.46 ± 0.56	1.57 ± 0.71
**EF (%)**	74.89 ± 12.19	73.11 ± 15.40	87.36 ± 7.86	87.23 ± 10.65

Data are presented as mean ± SD. * *p* < 0.05, ** *p* < 0.01 and *** *p* < 0.001 vs. Control; ^†^ *p* < 0.05 and ^††^ *p* < 0.01 vs. DOCA-salt. Number of animals per experimental group: 5.

**Table 2:** Primary and secondary antibodies used for immunofluorescence analysis. Primary and secondary antibodies are listed together with their host species, clonality, working dilution, manufacturer, and incubation conditions. The fluorophores conjugated to the secondary antibodies are also indicated.

**Primary Antibody**	**Host**	**Clonality**		**Dilution**	**Company**	**Incubation**
ET_A_	Rabbit	Polyclonal		1:500	Alomone (Israel)	
ET_B_	Rabbit	Polyclonal		1:500	Alomone (Israel)	
AVP	Rabbit	Polyclonal.		1:200	Merk Chemicals (Darmstadt, Germany)	
tTH	Rabbit	Polyclonal		1:500	Invitrogen (Carlsbad, CA,USA)	
GAP-43	Rabbit	Polyclonal		1:1000	Novusbio (Centennial, CO, USA)	Overnight at 4 °C
NF-κβ	Rabbit	Monoclonal		1:400	Cell Signaling (Danvers, MA, USA)	
**Secondary Antibody**	**Host**	**Fluorphobe**	**Primary Antibody**	**Dilution**	**Company**	**Incubation**
Rabbit	Goat	Alexa 488	ET_A_	1:250	Jackson ImmunoResearch (West Grove, PA, USA)	4 h at room temperature in darkness
	Goat	Alexa 488	ET_B_	1:250	Jackson ImmunoResearch (USA)	
	Goat	Alexa 405	AVP	1:400	Invitrogen (USA)	
Rabbit	Goat	FICT	tTH	1:500	Jackson ImmunoResearch (USA)	1.5 h at room temperature in darkness
	Mouse	TR	GAP-43	1:100	Santa Cruz Biotechnology (Dallas, TX, USA)	
	Goat	TR	NF-κβ	1:400	Santa Cruz Biotechnology (USA)	

**Table 3:** Primary and secondary antibodies used for Western blot analysis. Primary and secondary antibodies are listed together with their host species, clonality, working dilution, manufacturer, and incubation conditions.

**Primary Antibody**	**Host**	**Clonality**	**Dilution**	**Company**	**Incubation**
ANP	Mouse	Monoclonal	1:750	Santa Cruz Biotechnology (USA)	
BNP	Mouse	Monoclonal	1:750	Santa Cruz Biotechnology (USA)	
tTH	Rabbit	Polyclonal	1:1000	Sigma (USA)	
TH-psER40	Rabbit	Polyclonal	1:500	Invitrogen (USA)	
Col-1	Mouse	Monoclonal	1:500	Santa Cruz Biotechnology (USA)	
Col-3	Mouse	Monoclonal	1:500	Santa Cruz Biotechnology (USA)	
TLR-4	Mouse	Monoclonal	1:1000	Santa Cruz Biotechnology (USA)	Overnight at 4 °C
IL-1β	Mouse	Monoclonal	1:750	Santa Cruz Biotechnology (USA)	
IL-6	Mouse	Monoclonal	1:500	Santa Cruz Biotechnology (USA)	
TNFα	Mouse	Monoclonal	1:1000	Santa Cruz Biotechnology (USA)	
iNOS	Rabbit	Polyclonal	1:500	BD Biosciences (Milpitas, CA, USA)	
IL-10	Mouse	Monoclonal	1:750	Santa Cruz Biotechnology (USA)	
GPDH	Mouse	Monoclonal	1:30,000	Invitrogen (USA)	
**Secondary Antibody**	**Host**	**Primary Antibody**	**Dilution**	**Company**	**Incubation**
		ANP	1:3000		1 h.30 min at Room Temperature
		BNP	1:3000		
		Col-1	1:2000		
		Col-3	1:2000		
		TLR-4	1:2000		
Mouse	Goat	IL-1β	1:10,000	Bio-Rad (Hercules, CA, USA)	
		IL-6	1:1500		
		TNFα	1:5000		
		IL-10	1:5000		
		GPDH	1:40,000		
		tTH	1:10,000	Merck Millipore (Burlington, MA, USA)	1 h.30 min at Room Temperature
Rabbit	Goat	TH-psER40	1:10,000		
		iNOS	1:2000		

**Table 4:** Primer sequences used for quantitative reverse transcription real-time PCR (RT-qPCR) analysis. Forward and reverse primer sequences and the corresponding expected amplification product sizes are indicated for each gene.

Gene	Primer Sequences (5′-3′)	Product Size (bp)
ANP	Forward: 5′-GAGTGAGCCGAGACAGCAAA-3′	170
	Reverse: 5′-CAGGTGGTCTAGCAGGTTCTTG-3′	
BNP	Forward: 5′-TAGTTCAAGCTGCTTTGGGC-3′	250
	Reverse: 5′-CACTGTGGCAAGTTTGTGC-3′	
B-MHC	Forward: 5′-ACAGGAAGAACCTACTGCGA-3′	264
	Reverse: 5′-TCCTTAGGGTTGGGTAGCAC-3′	
PRO-COL 1	Forward: 5′-AGGGACACAGAGGTTTCAGT-3′	183
	Reverse: 5′-CATCGTTACCACGAGCACC-3′	
PRO-COL 3	Forward: 5′-TTGGCACAGCAGTCCAATGTA-3′	164
	Reverse: 5′-GGGCAGTCTAGTGGCTCATC-3′	
TLR-4	Forward: 5′-CTCACAACTTCAGTGGCTGGATTTA-3′	177
	Reverse: 5′-GTCTCCACAGCCACCAGATTCTC-3′	
TNFα	Forward: 5′-ATCCGAGATGTGGAACTGGC-3′	472
	Reverse: 5′-AAATGGCAAATCGGCTGACG-3′	
IL-1β	Forward: 5′-GCCAACAAGTGGTATTCTCCA-3′	244
	Reverse: 5′-GGGAACTGTGCAGACTCAAA-3′	
IL-6	Forward: 5′-TGGTCTTCTGGAGTTCCGTT-3′	176
	Reverse: 5′-GGAGAGCATTGGAAGTTGGG-3′	
NF-κβ	Forward: 5′-CGCGGGGACTATGACTTGAA-3′	163
	Reverse: 5′-AGTTCCGGTTTACTCGGCAG-3′	
GAPDH	Forward: 5′-CTCTGCTCCTCCCTGTTCTA-3′	250
	Reverse: 5′-ATTCTCAGCCTTGACTGTGC-3′	

## Data Availability

The data presented in this study are available in the article.
